# Salivary biomarkers: novel noninvasive tools to diagnose chronic inflammation

**DOI:** 10.1038/s41368-023-00231-6

**Published:** 2023-06-29

**Authors:** Paola Dongiovanni, Marica Meroni, Sara Casati, Riccardo Goldoni, Douglas Vieira Thomaz, Nermin Seda Kehr, Daniela Galimberti, Massimo Del Fabbro, Gianluca M. Tartaglia

**Affiliations:** 1 Medicine and Metabolic Diseases, Fondazione IRCCS Cà Granda Ospedale Maggiore Policlinico, Milan, Italy; 2 Department of Biomedical, Surgical and Dental Sciences, University of Milan, Milan, Italy; 3 Department of Electronics, Information and Bioengineering (DEIB), Politecnico di Milano, Milan, Italy; 4 Istituto di Elettronica e di Ingegneria dell’Informazione e delle Telecomunicazioni, CNR, Pisa, Italy; 5 Laboratory of Medicinal Pharmaceutical Chemistry, Faculty of Pharmacy, Federal University of Goiás, Goiânia, GO Brazil; 6 Department of Chemistry, İzmir Institute of Technology, Gülbahçe Kampüsü, Urla İzmir, Turkey; 7 Neurology-Neurodegenerative Diseases, Fondazione IRCCS Cà Granda, Ospedale Maggiore Policlinico, Milan, Italy; 8 UOC Maxillo-Facial Surgery and Dentistry Fondazione IRCCS Cà Granda, Ospedale Maggiore Policlinico, Milan, Italy

**Keywords:** Diagnostic markers, Biological techniques, Predictive markers

## Abstract

Several chronic disorders including type 2 diabetes (T2D), obesity, heart disease and cancer are preceded by a state of chronic low-grade inflammation. Biomarkers for the early assessment of chronic disorders encompass acute phase proteins (APP), cytokines and chemokines, pro-inflammatory enzymes, lipids and oxidative stress mediators. These substances enter saliva through the blood flow and, in some cases, there is a close relation between their salivary and serum concentration. Saliva can be easily collected and stored with non-invasive and cost-saving procedures, and it is emerging the concept to use it for the detection of inflammatory biomarkers. To this purpose, the present review aims to discuss the advantages and challenges of using standard and cutting-edge techniques to discover salivary biomarkers which may be used in diagnosis/therapy of several chronic diseases with inflammatory consequences with the pursuit to possibly replace conventional paths with detectable soluble mediators in saliva. Specifically, the review describes the procedures used for saliva collection, the standard approaches for the measurement of salivary biomarkers and the novel methodological strategies such as biosensors to improve the quality of care for chronically affected patients.

## Introduction

Inflammation is a process that the host enacts to defend itself against toxins, bacteria, viruses, tissue damage, metabolic stress by recruiting immune and non-immune cells. Thus, in a physiological context inflammation is protective and once the insult is eradicated several mechanisms intervene and lead to a process named “resolution of inflammation”.^1^ A prolonged inflammatory status may become chronic and pathological when the regulatory events which promote the resolution are lost.

Both acute and chronic inflammation share several phases: increased blood flow to the site of inflammation, higher capillary permeability to allow even larger molecules to cross the endothelium, recruitment of leukocytes from the capillaries to the surrounding tissue and finally the release of mediators by the latter, including cytokines, chemokines, markers of oxidative stress (as superoxide), enzymes (i.e. metalloprotease) and lipid mediators as prostaglandins and leukotrienes.^2^

The majority of chronic diseases are preceded by a chronic low-grade inflammation. Hence, conceivable biomarkers for the early assessment of disorders encompass acute phase proteins (APP), cytokines and chemokines, pro-inflammatory enzymes, and oxidative stress mediators. Among the most common chronic diseases, heart disease, cancer, obesity, and type 2 diabetes (T2D) constitute the leading causes of disability and death in the United States.^3,4^ Given the widespread manifestations, the number of hospitalization and the mortality rate, these conditions represent a huge socio-economic burden. Indeed, the affected subjects require ongoing medical attention from the first symptoms to the management of therapeutic options.

Therefore, the identification of forefront diagnostic tools is essential to establish preventive approaches and targeted pharmacological interventions with the purpose of minimizing the risks, distress and ultimately the costs of chronic diseases. In this context, the possibility to exploit salivary biomarkers as tool to detect systemic disorders may constitute an intriguing opportunity to implement the strategies to diagnose and follow-up patients affected by chronic disorders, limiting the risks related to more invasive procedures.

Saliva is an exocrine secretion of the salivary glands mainly composed of water (99%), but it also contains electrolytes, proteins, lipids, and enzymes. Contaminants such as bacteria, epithelial cells, gingival crevicular fluid and food debris are also detectable in saliva.^5^ Proteins and other substances enter saliva through the blood flow and in some case, there is a close relation between their salivary and serum concentration. Indeed, salivary glands are highly vascularized and there is an exchange of compounds which pass by passive diffusion or active transport from blood to saliva and *viceversa*. To this regard, recent studies have reported the diagnostic utility of saliva to detect cardiovascular diseases (CVD), systemic and local inflammation, endocrinological and metabolic disorders.^6^ Salivary lipids are mostly secreted by the major salivary glands, but some lipids like cholesterol and some fatty acids (FAs) diffuse directly from serum into saliva.^7^ Therefore, the use of saliva as an alternative diagnostic tool is advisable since its collection is non-invasive and possibly stress-free. In addition, no differences were found between men and women in salivary composition although the literature is still contradictory.^6,8–11^

Furthermore, a large amount of saliva can be easily collected and stored with non-invasive and cost-saving procedures.^12,13^ Therefore, the opportunity to detect and quantify biomarkers in salivary samples becomes highly attractive for research, clinical, and unobtrusive proactive healthcare applications, with the purpose to early diagnose chronic diseases and to allow a continuous disease monitoring.

To this purpose, the present review aims to discuss the advantages and challenges of using cutting-edge techniques to discover salivary biomarkers which may be used in diagnosis/therapy of several chronic diseases with inflammatory consequences, with the pursuit to possibly replace conventional paths with detectable soluble mediators in saliva.

## Modulators of the inflammatory status

The inflammatory milieu is firstly affected by the triggering event which in turn may determine the recruitment of different numbers and types of immune cells and, accordingly, a diversified release of proinflammatory mediators. However, several factors can cause dynamic alterations in serum biomarkers of inflammation including age, presence of obesity, gender, diet, smoking, genetics, drug consumption and gut microbiota.

### Age

Aging is featured by an increase in serum levels of cytokines and acute phase proteins (APPs) due to a low-grade inflammatory status which may be induced by increased visceral adiposity, declined function of sex hormones, genetics, neurodegenerative disorders as Alzheimer’s disease (AD) or cardiovascular complications. Interleukin-6 (IL-6) levels are increased in elderly subjects to the point that it has been dubbed a cytokine for gerontologists.^14^ Similarly, it has been reported that circulating tumor necrosis factor alpha (TNFα), IL-1 as well as C-reactive protein (CRP), α1-acid glycoprotein and fibrinogen increase with age.^15^ In healthy, elderly populations, high circulating levels of TNFα and IL-6 predict mortality, regardless of comorbidities, whereas in cohorts of frail, older individuals, these cytokines may represent risk factors for atherosclerosis, T2D, AD, thromboembolic complications, and are associated with sarcopenia and muscle loss.^16,17^

Higher levels of IL-18, a linked IL-1 pro-inflammatory cytokine, IL-2, IL-17 and IL-12 have been found in elderly, associated with CVD, stroke, type 1 diabetes, AD and osteoarthritis.^18–21^

Finally, IL-17 and IL-8 (CXCL8) promote inflammation, recruitment and activation of neutrophils and increased levels have been found in autoimmune diseases such as systemic lupus erythematosus, inflammatory bowel disease and psoriasis.^22^ Moreover, lipopolysaccharide (LPS)-stimulation of leukocytes from elderly individuals induces IL-8 release and the latter has been also suggested as a possible longevity factor in centenarians.^23^

### Adiposity

Obesity is associated with low-grade chronic inflammation. Visceral fat produces several proinflammatory cytokines (TNFα, IL-1, IL-6) and chemokines (MCP-1) and it has been described that also ectopic sites of adipose tissue such as those localized in liver, heart or muscle may contribute to the release of pro-inflammatory mediators also independently of body fatness.^24^

Elevated CRP levels were found in overweight (body mass index [BMI], 25–29.9 kg·m^−2^) and obese (BMI, ≥30 kg·m^−2^) individuals compared to normal weight ones and regardless of age.^25^ Moreover, it has been suggested that TNFα pathway could be involved in the regulation of circulating leptin, whose levels are elevated in obese subjects.^26^ Conversely, it has been described a tendency for reduced adiponectin levels in obese subjects possibly mediated by TNFα.^27^

The state of adiposity is closely correlated with physical inactivity, which leads to an increase in visceral fat. It has been demonstrated that aerobic exercise leads to reduced circulating IL-6 and CRP, as shown in a study on 2 120 Finnish participants where the levels of the latter were positively associated with obesity indices and inversely related to physical activity.^28^ Finally, in a large intervention study CRP concentration diminished by 41% in subjects who performed physical activity compared to inactive ones.

### Gender

Clinical findings indicate that inflammatory responses differ across sexes, although data are still conflicting. In the CoLaus study which assessed the determinants of cytokines and APPs levels in a Caucasian population made of 2 884 men and 3 201 women, male gender was independently and positively related to IL-6 and TNFα levels whereas for IL-1 and CRP no associations were found.^29^

In another small study, which enrolled 15 healthy women and 20 healthy men, higher concentrations of IL-12, IL-1β, and TNFα and lower levels of IL-2 were found in males compared to women.^30^ Differences in lipid mediator levels have been observed between males and females in multiple diseases, such as prostaglandins (PGs) in T2D,^31^ linoleic acid-derived lipid mediators in chronic obstructive pulmonary disease,^32^ docosahexaenoic acid (DHA) in AD,^33^ and lipoxin A4 (LXA4) in metabolic syndrome.^34^

### Smoking

Several studies have assessed a close relationship between smoking and chronic inflammation. Cigarettes contain oxidative molecules (superoxide, hydrogen peroxide, nitrogen oxides) which drive oxidative stress thus leading to an inflammatory response, several toxins with immunomodulatory effects and also trace amounts of microbial cell components, including bacterial LPS.^35^

It has been definitively established that IL-1β, IL-6, CRP, and fibrinogen are sensitive biomarkers for cigarette smoke-induced inflammation.^36^ In addition, it has been shown that the imbalance between oxidants and antioxidants resulting from exposure to tobacco smoke leads to oxidative stress, increased mucosal inflammation, enhanced release of IL-8, IL-6, and TNFα and to the recruitment of macrophages and neutrophils.^37^

Concerning lipid biomarkers related to smoking status, arachidonic acid (AA) derived lipoxygenase (LOX)-metabolites, which are potent pro-inflammatory mediators leading to tissue destruction in periodontal inflammation,^38^ are significantly increased in smokers *vs*. non-smokers. In addition, 8-iso-prostaglandin F_2α_ (8-iso-PGF_2α_) excretion exhibits dose-dependent increments in individuals who smoke cigarettes or consume alcohol.^39,40^ In contrast, 6-oxo- prostaglandin F_1a_ (PGF_1a_), prostaglandin I_2_ (PGI_2_) and prostaglandin F_2a_ (PGF_2a_) were significantly decreased in smokers *vs*. non-smokers. There are contradictory results in prostaglandin E_2_ (PGE_2_) synthetic rate, cigarette smoking and bone loss.^41,42^

### Epigenetics and genetics

Emerging evidence suggests that epigenetics (DNA methylation, histone acetylation/deacetylation and microRNA (miRNA) expression) may contribute to the pathophysiology of inflammatory processes.^43^ Epigenome-wide association studies (EWAS) have reported several epigenetic changes related to serum inflammatory markers suggesting a global hypomethylation of the genome during inflammation.^44^ Indeed, miR-126, miR-132, miR-146, miR-155, and miR-221 have recently emerged as important transcriptional regulators of TNFα, IL-8, MCP-1, IL-6, and adhesion molecules.^45^

Epigenetic changes may also affect the risk of chronic inflammatory diseases, including obesity, T2D, CVD, cancer, and neurological disorders. Prats-Puig et al. reported that 15 specific circulating miRNAs were significantly deregulated in prepubertal obesity, including a downregulation of miR-221 and miR-28-3p and an upregulation of miR-486, miR-142-3p, miR-130b, and miR-423-5p in plasma.^46^ It has been demonstrated that the inhibition of miR-153 prevents hyperglycemia in *db/db* mice, thus suggesting that it may be a promising therapeutic target for the treatment of inflammation-associated diabetes.^47^ Jiang and colleagues demonstrated that steatotic hepatocyte-derived extracellular vesicles (EVs) promote endothelial inflammation and facilitate atherogenesis by miR-1 delivery, KLF4 suppression and NF-κB activation by exploiting apolipoprotein E (*ApoE*)-deficient mice.^48^ Finally, several miRNAs regulate the expression of genes involved in AD-related oxidative stress^49^ and their levels may be influenced by transcription factors, among which NF-kB thus modulating inflammation and cancer.^50^

Genetic polymorphisms, especially in genes encoding molecules of the host defense system, such as cytokines, influence susceptibility to chronic inflammation. The −174 G/C genetic variant in the promoter region of IL-6 gene has been related to reduced gene expression and circulating levels of IL-6 and it has been described an association between this polymorphism, AD and also coronary artery disease (CAD).^51,52^ The G > A nucleotide substitution at position 308 in the TNFα promoter directly affects TNFα expression and has been associated with several inflammatory conditions as liver disease, primary sclerosing cholangitis, biliary cirrhosis, Crohn’s disease, rheumatoid arthritis and CAD.^53^ Finally, the 1082 G > A *IL-10* and −308G > A *TNFα* variations have been associated with lower circulating levels of the anti-inflammatory IL-10 and with higher serum TNFα in 72 centenarians compared to controls, respectively.^54^

## Methodological approaches for the detection of salivary biomarkers

### Methods for saliva sampling and collection

Saliva has largely been disregarded in the past due to the presence of several food contaminants in its biochemical profile, as well as paucity of demonstration of correlations between salivary and blood markers.^55^ However, several studies have recently highlighted how saliva well correlates with blood markers, thus representing a promising alternative for noninvasive diagnostics,^56^ as it is the case with glucose^57^ and cortisol.^58^ Among others, IL-6, an inflammatory marker, also shows a significant correlation between blood and saliva^59^ (Table 1).

**Table 1:** Inflammatory biomarkers detected in serum and saliva in healthy subjects

Biomarkers		Serum concentration	Saliva concentration
Cytokines/chemokines	TNFα	60–90 pg·mL^−1^	20–45 pg·mL^−1^
	IL-6	0–44 pg·mL^−1^	0.5–34 pg·mL^−1^
	IL-1α	31.4 pg·mL^−1^	361 pg·mL^−1^
	IL-1β	0.5–20 pg·mL^−1^	40.5–494 pg·mL^−1^
	IL-2	21 pg·mL^−1^	4.3–10.3 pg·mL^−1^
	IL-4	10–20 ng·L^−1^	15–25 ng·L^−1^
	IL-5	2.6–13 pg·mL^−1^	0–3.5 pg·mL^−1^
	IL-7	6.5 pg·mL^−1^	8.3 pg·mL^−1^
	IL-8	6.8–39 pg·mL^−1^	150–400 pg·mL^−1^
	IL-10	0.5–2.9 pg·mL^−1^	0.5–5.1 pg·mL^−1^
	IL12p70	2–10.5 pg·mL^−1^	19.2 pg·mL^−1^
	IL-13	17 pg·mL^−1^	0.7 pg·mL^−1^
	IL-15	65.5–170 ng·L^−1^	0–8.7 pg·mL^−1^
	IL-17a	15–40 pg·mL^−1^	5–10 pg·mL^−1^
	IFNγ	20–42 pg·mL^−1^	28 pg·mL^−1^
	CCL2 (MCP1)	6–70 pg·mL^−1^	125 pg·mL^−1^
	CCL3 (MIP1α)	5.2 pg·mL^−1^	2.3 pg·mL^−1^
	eotaxin (CCL11)	35–50 pg·mL^−1^	5.2–6.2 pg·mL^−1^
	TGFβ	1.8–26 ng·mL^−1^	5.4–30 ng·mL^−1^
Proinflammatory enzymes	MMP8	5.7–19 ng·mL^−1^	2.5–309 pg·mL^−1^
	MMP9	215–608 ng·mL^−1^	50–100 ng·mL^−1^
	TIMP1	305–342 g·L^−1^	1.5–3 pg·mL^−1^
	TIMP2	100–200 ng·mL^−1^	2–3.5 ng·mL^−1^
	Carboxyterminal telopeptide of type I collagen (ICTP)	3.5–4.5 ng·mL^−1^	15.2 ng·mL^−1^
Antioxidant markers	8-Hydroxy-2′-deoxyguanosine (8-OHdG)	121–200 ng·L^−1^	6.5–7.5 ng per 1 mg albumin
	Malondialdehyde (MDA)	0-29-0.98 mmol·L^−1^	0.85–4.31 mmol·L^−1^
	Uric acid	0.5–1.5 mg·dL^−1^	2.8–4 mg per 1 mg albumin
	Glutatione peroxidase (GPX)	196–477 U·L^−1^	17–39 U per 1 mg albumin
	TAC (total antioxidant capacity)	(1.92 ± 0.34) mmol trolox equivper L	1.1–1.5 nnol per 1 mg albumin
	Superoxide dismutase (SOD)	0.78–1.48 U·mL^−1^	0.6–1.53 U·mL^−1^
	Glutathione (GSH)	1.91–4.41 μmol·L^−1^	1.1 (0.1–3.3) μmol·L^−1^
	Mieloperossidase (MPO)	30–40 ng·mL^−1^	(0.40 ± 0.16) μmol·L^−1^
	4-hydroxynonenal (4-HNE)	0.5–2 μg·mL^−1^	0–0.15 μg·mL^−1^
Acute phase proteins	C-reactive protein (CRP)	0.1–10 mg·L^−1^	0–472 pg·mL^−1^
	Serum amyloid A (SAA)	15–35 mg·L^−1^	3.1–423 U·mL^−1^
	Haptoglobin (Hp)	50–220 mg·dL^−1^	451–1 457 μg·L^−1^
	C3	1–3 mg·L^−1^	0–2 mg·mL^−1^
	Alpha1antitrypsin (AAT)	0.9–1.75 g·L^−1^	2–2 271 ng·mL^−1^
	IL-1ra	350–700 ng·L^−1^	3 700 pg·mL^−1^
	Ferritin	10–250 ng·mL^−1^	147–191 mg·L^−1^
	Cortisol	5–23 mg·dL^−1^	3–19 mg·L^−1^
	Lipopolysaccaride (LPS)	0.1–10 mg·mL^−1^	4.2–10.1 mg·mL^−1^
Adipokines	Leptin	10 ng·mL^−1^	21–42 pg·mL^−1^
	Adiponectin	0-5-30 μg·mL^−1^	29 mg·dL^−1^

Although the procedures for saliva collection and storage have been already described in other reviews,^60,61^ it is important to contextualize these methods in the field of inflammatory markers, delineating best practices for a correct collection, handling, and biobanking. Moreover, although saliva receives increasing consideration as a potential sample for target diseases,^62^ it is still warranted a comparison with the current diagnostic methodologies to evaluate inflammatory markers in blood.

The gold standard method of saliva collection is “passive drool”, that consists in saliva accumulation in the mouth and then let it flow through specific straws that collect the pooled saliva for a predetermined amount of time.^61^ Saliva can be easily sampled by either health professionals or people without previous medical training.^63,64^ Moreover, there are numerous standardized protocols for oral sampling, and saliva collection can be easily automated by means of wearable modules.^65,66^ Nevertheless, it must be remarked that saliva is a complex mixture of fluids from distinct microenvironments, such as gingival crevices,^67^ which may present a particular microbial population that often differs from that of other oropharyngeal regions.^68^

**Fig. 1:**
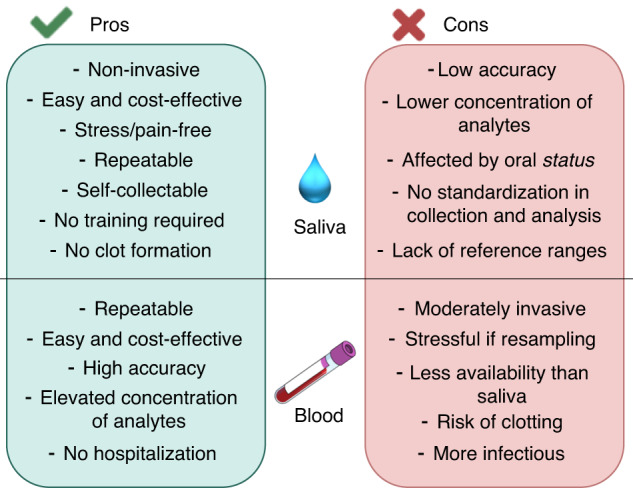
Pros and Cons of the use of saliva or blood for the detection of clinically relevant analytes

**Fig. 2:**
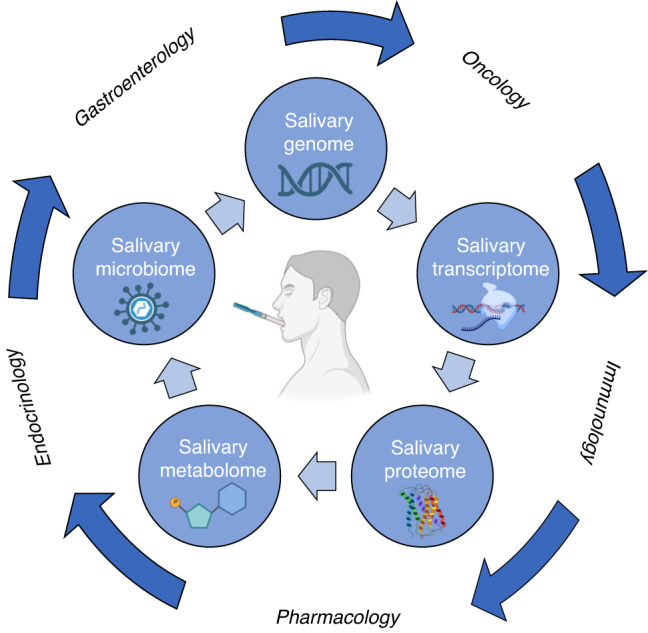
Possible applications and clinical utility of saliva, as biological fluid for omics-based studies

Other collection procedures include spitting, chewing, and swab-based methods. Saliva collected through spitting only involves specific salivary glands such as the submandibular and minor salivary ones and it is thus less preferred, whereas chewing, usually performed through the use of wax, allows the collection of stimulated saliva mainly from parotid glands. The latter is currently the recommended medium to measure the concentration of CRP in saliva,^69^ while as concern the other inflammatory markers, unstimulated saliva remains the most preferred fluid. Lastly, swab-based methods have been largely used in SARS COVID-19 tests and different studies report how this method can be particularly suited to perform salivary analysis in children.^70^ However, it has also been demonstrated how immunoassay tests showed a high deviation in the concentration of salivary markers due to the use of cotton-based swabs^71^ (Figs. 1–2).

### Standard approaches for the detection of salivary biomarkers

The assays that are currently used for salivary analysis of inflammatory markers require the samples to be sent to centralized laboratories, while there are only few examples of commercial products that allow for point of care salivary diagnostics. Costs and time to response are the two parameters mainly taken into consideration in evaluating these procedures.

The most widespread immunoassay, possibly representing the gold standard for many examinations is the enzyme linked immunosorbent assays (ELISA), which allows the detection of target antigens through the use of plates functionalized with antibodies. ELISA kits are available for the detection of several targets, including inflammatory markers such as cytokines,^72^ both in centralized laboratories and on the market (i.e. Salimetrics). A panel of biomarkers can be screened by using multiplexed technologies where their concentration is simultaneously evaluated, providing more comprehensive results and faster lead times.

Since ELISA is used for both blood and saliva tests, it is of utmost importance to compare results coming from a novel tool with it. Studies which describe the use of sensors and innovative devices for salivary screening, do not always report a validation against standardized procedures,^66,73,74^ thus limiting the applicability of the obtained results for clinical trials.

### Novel perspectives to analyze salivary biomarkers

To overcome salivary sample complexity, many researchers have investigated protocols to separate non-biomarker proteins and carbohydrates from raw saliva, thereby yielding pre-treated testing material that could be easier to analyze.^75–77^ This strategy has been nonetheless pursued due to the issue of viscosity, that could hinder the application of microfluidic arrays on raw untreated saliva. However, even if this treatment may minimize the effect of potential interferents, it might also compromise the concentration of target biomarkers^78^ as mucin which is an inflammatory biomarkers for periodontitis^79^ and matrix metalloproteinases,^80^ which can be lost from the sample upon membrane filtration procedures. Moreover, the removal of biomarkers from the sample poses a major hindrance if the molecules to be detected form complexes with other macromolecules and precipitate, thus decreasing their final concentrations in pre-treated samples.^81^ The opportunity to employ untreated or undiluted saliva is highly sought after by scientists worldwide, and great attention has been given to the development of highly selective and sensible biosensing platforms.

In order to increase the specificity and sensibility of analytical strategies to determine inflammatory biomarkers, several researchers have highlighted the possibility of using surface-modified substrates as biorecognition elements.^82,83^ In this regard, several materials (carbon-or gold-based materials) have been discussed as promising alternatives for biosensor development.^84^ The benefits of carbon-based materials in biosensing are numerous, owing to many allotropic forms bearing highly conductive sp2 lattices,^85^ as well as offering adequate stability for the anchoring of coordination complexes and construction of metalorganic frameworks that are capable of enhancing analytic signal acquisition.^86^ Moreover, even though advanced carbon materials such as graphene may be expensive, the methods for their production are in constant development, and this resource is abundant in nature as opposed to precious metals. On the other hand, gold-based substrates offer simplicity and an easier function as their main benefit.^87^ It is well reported that thiolate compounds show thermodynamic feasibility to spontaneously assemble monolayers on gold substrates.^88^ This phenomenon, named self-assembled monolayer formation has been extensively explored in the crafting of biosensing strategies to determine inflammatory biomarkers in saliva and other biological fluids, and can be easily performed on room temperature without the need of extreme conditions.^89,90^

Electrochemical,^91^ optical^92^ and acoustic transduction^93^ represent the most diffuse signal transduction technologies used for the development of biosensors for the detection of salivary inflammatory biomarkers. Electrochemical technologies have as analytical principle the inherent electrical properties of matter, by measuring changes in electrical charge, current, potential and resistance.^91^ Overall, this method has been the most used by researchers in the development of label-free biosensing platforms for oral applications, due to the versatility, low cost and high portability.^91^ On the other hand, methods based on optical signal transduction rely on shifts in spectra absorption, reflection and refraction and they often employ spectrophotometry, spectroscopy, colorimetry and surface-plasmon resonance.^83^ These techniques offer high sensibility and selectivity, and similarly to electrochemical transduction, also allow the development of either label-free or sandwiched biosensing strategies. In regard to acoustic signal transduction, this approach is a recent trend in biosensing technologies, and has attracted much attention due to the possibility of refining the signal acquisition and attaining very low limits-of-detection.^94^ This technology mostly bases on the dependence of frequency and dissipation of mechanical disturbances, which can be generated by piezoelectric materials serving as sensing substrates.^94^ However, owing to its novelty and reliance on high frequencies, there are still limitations regarding its point-of-care application.^94^

**Fig. 3:**
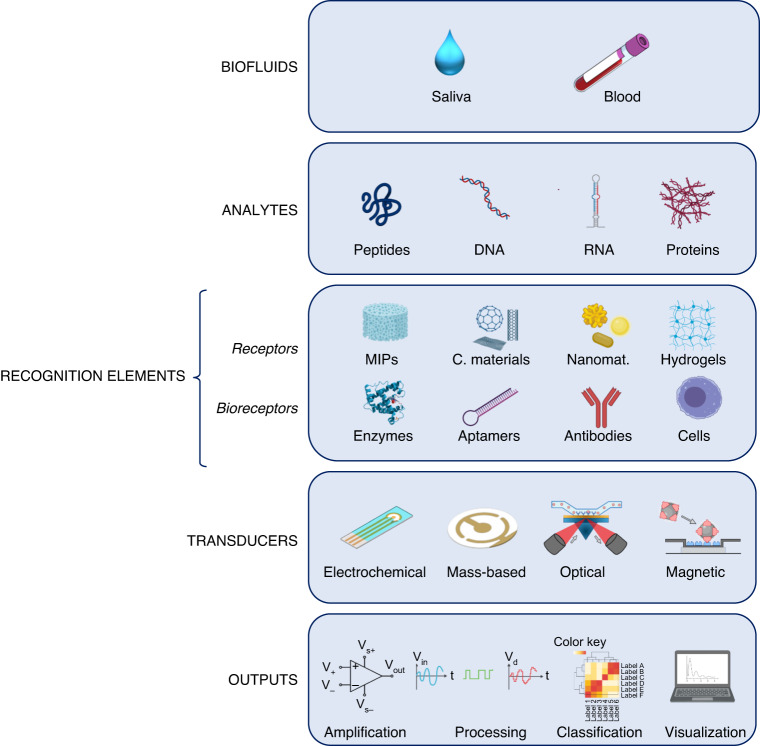
Saliva and blood as biological fluids to exploit biosensors to detect peptides, DNA, RNA and proteins. Schematic representation of the components of biosensors and workflow

In order to improve the specificity and the sensitivity of biosensors, molecularly imprinted polymer (MIP) technology is implemented onto the biomaterial surfaces. In MIP, specific functional monomers are polymerized in the presence of the target molecule, e.g., a biomarker. The target molecule is then removed, leaving a polymer matrix that has recognition cavities that are complementary to the target molecule in terms of size, shape, and functionality. In this way, MIP specifically rebinds the target molecule and reduces the effect of potential interferents on false positives and thus enhance safe data generation.^95^ MIPs have been successfully applied not only for small molecules recognition but also for biomacromolecules, such as proteins.^96^ MIPs are used in the design of MIPs-based biosensors, due to their higher stability, specificity, and reusability than biological receptors. However, industrial application of MIP-based biosensors is limited due to their lack of reproducible preparation and stability on the sensor substrate, as well as the limited slow diffusion of analyte into the cavities and binding sites (Fig. 3).

**Fig. 4:**
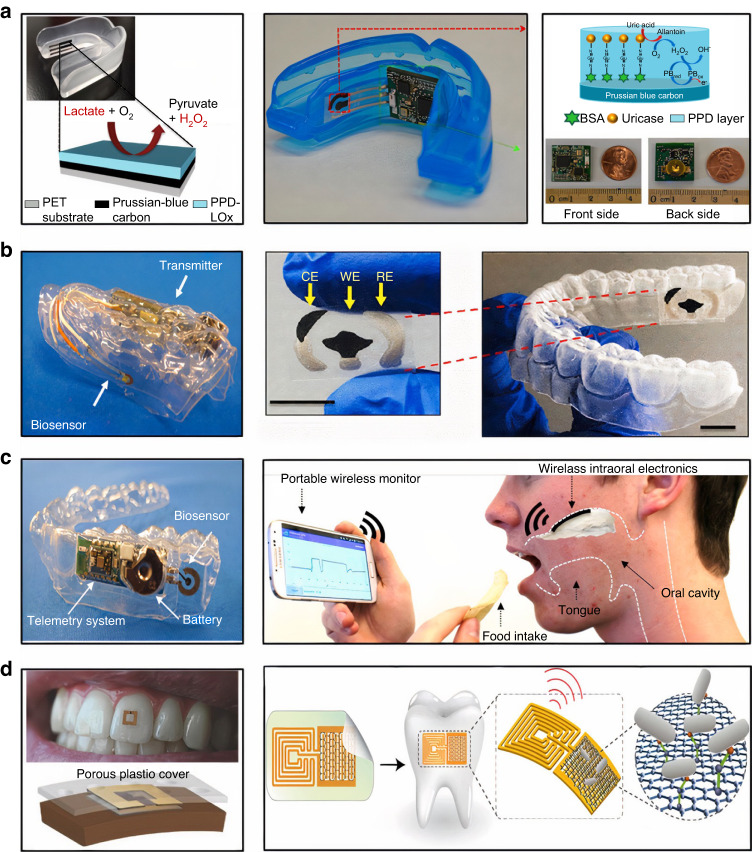
Summary of wearable intraoral biosensing platforms for non-invasive salivary analysis. Lactate biosensor on a mouthguard (**a**, on the left). Reproduced with permission from Kim et al..^86^ Mouthguard with biosensor and integrated electronics for real-time uric acid detection (**a**, on the right). Reproduced with permission from Kim et al..^86^ Mouthguard with screen printed electrodes for N-Carboxymethyl lysine detection (**b**). Reproduced with permission from Ciui et al..^177^ Glucose biosensing telemetry system (**c**, on the left), reproduced with permission from Arakawa et al.,^178^ and a hybrid flexible bioelectronic platform for sodium monitoring (**c**, on the right). Tooth mounted hydrogel radiofrequency biosensor (**d**, on the left), reproduced with permission from Tseng et al.^179^ and graphene-based biosensor for pathogen’s detection (**d**, on the right), reproduced with permission from Mannoor et al.^99^

The rapid advances in nanotechnology, microelectronics, and Internet of Things enable the development of wearable biosensors that can be positioned in the oral cavity for the detection of salivary biomarkers.^74^ Such biosensors communicate with computers/smartphones wirelessly and enables the online data analysis. Data transfer is generated through WiFi and BlueTooth Low Energy. For example, Kim et al. reported an integrated wireless mouthguard amperometric biosensor that enables the non-invasive monitoring of salivary uric acid levels.^97^ Lee at al. described a wireless intraoral device capable of a real-time recording of sodium detection.^98^ Mannoor et al. fabricated a graphene-based wireless biosensor for remote monitoring of respiration and bacteria detection in saliva.^99^ Tseng at al. demontrated the wireless monitoring of oral cavity and food consumption by a radiofrequency-trilayer dielectric sensor^100^ (Fig. 4).

Technological developments have provided a growing improvement in methods that may be exploited to measure disease biomarkers although the detection of the latter is challenging due to the high inter-individual variability. Therefore, machine learning models have been developed to identify signatures in multiple circulating biomarkers for specific diseases. Machine learning algorithms enable full automation and allow to analyze large datasets in a short period of time by reducing false positives that lead to incorrect diagnosis, and thus saving clinicians’ time for data analysis. Currently, machine learning methods including Random Forests or Gradient Boosted Trees to deep learning have been increasingly applied to identify biomarkers from body fluids for non-invasive disease diagnosis.^101–104^

Despite the scientific and technological advances, an early and non-invasive biomarker detection is still limited by current biosensors. In particular, the co-detection of different biomarkers that characterize a specific disease, by a single biosensor, is still unripe. Therefore, multiplex technologies for advanced biosensor manufacturing and biomarker detection are needed to improve the quality of patient care and to reduce the costs through the early assessment and diagnosis of chronic diseases.

## Salivary biomarkers: an attractive way to approach screening and monitoring

To date, soluble markers of chronic inflammation have been assessed in blood. However, recent findings have demonstrated that inflammatory mediators may be detectable also in saliva which has drawn a growing attention as biological fluid especially due to its stress-free and non-invasive collection.

### Acute phase proteins (APPs) as salivary biomarkers

Chronic subclinical inflammation and tissue injuries are associated with the secretion of acute phase proteins, such as haptoglobin (Hp), CRP, Alpha 1-antitrypsin (A1AT), fibrinogen and ferritin, mainly from the liver into the bloodstream.^105^ Their concentrations progressively increase with the tissue damage and then, these substances passively diffuse through the porous capillaries, are actively transported into saliva or may also diffuse between acinar cells.^106^ Some of them also directly derive from salivary glands.^107^ Abnormal serum levels of APPs are reported in several disorders, among which myocardial infarction, T2D, insulin resistance (IR), inflammatory bowel disease, psoriasis, and cancer. In detail, APPs are stratified in positive and negative ones, according to their up- or downregulation during inflammation.^108^

Among the positively regulated, CRP has been indicated as a predictor of primary and secondary adverse cardiovascular events, participating in atherogenesis whereby mediating the recruitment of innate immunity and the activation of complement pathway.^109^ Although it has a high molecular weight, CRP can enter into the oral cavity, through plasma exudates of systemic origin from gingival crevicular fluid (GCF).^110,111^ It has been demonstrated that CRP levels in saliva moderately correlated with those in serum samples and with systemic inflammation, postulating this *via* as a useful strategy to assess various inflammatory conditions.^112,113^ Indeed, CRP is synthetized only by the liver, and it is not produced locally in the mouth. Thus, its salivary levels may more accurately reflect the systemic inflammatory *status*, compared to cytokines and chemokines, that are modulated also by oral pathologies.^114^ For the same reason, fibrinogen concentrations assessed in saliva are representative of the blood protein content albeit with low levels and possible contaminations by ulcerated gingival epithelium.^114^ However, it seems to own reliable clinical utility to detect tuberculosis and polycystic ovarian syndrome (PCOS).^115,116^

Another molecule that has been pointed out as possible salivary biomarker of subclinical inflammation is haptoglobin (Hp),^117^ and it has been studied together with the cortisol hormone. Indeed, in stressful situations, the activation of the hypothalamic-pituitary-adrenal (HPA) axis is proven by the release of cortisol by adrenal cortex into the bloodstream within few minutes. A significant elevation of both Hp and cortisol has been identified in a model of experimentally induced systemic inflammation by an LPS challenge^117^ and salivary and blood concentrations of the latter have been found to be strictly correlated.^118,119^ Finally, the assessment of salivary A1AT levels may be used for monitoring effectiveness of oncological interventions.^120^

### Salivary biomarkers of inflammation

The feasibility to estimate the individual inflammatory *status* from saliva opens the route to unraveled approaches to the diagnosis and management of several inflammatory disorders and the possibility to non-invasively assess the response to acute stressors.^121^ However, salivary measurements often show inconsistencies, mainly due to various methodological applications, handling technique and timing of collection. Moreover, the degree of translatability of blood-based inflammatory markers on saliva-based ones and the pathological range of concentration for each salivary biomarker remain to be defined. Indeed, the impact of the fluctuations, due to local inflammatory milieu, oral mucosal immunity, and interruptions in gingival integrity on the content of these mediators in saliva needs to be taken into account. Therefore, standard procedures of collection and preservation are largely recommended for the utilization of saliva as a reliable diagnostic medium.^59^ Nevertheless, it has been established that a broad variety of interleukins (IL-1β, IL-2, IL-4, IL-6, IL-10 etc.), TNFα and pro-inflammatory enzymes, involved in matrix remodeling such as metalloproteinases and their inhibitors, may be quantified both in saliva and serum samples. For example, IL-1β, a cytokine released from macrophages and non-immune cells in the context of inflammation, participates to innate immune response, whereby promoting the secretion of IL-6 and TNFα. The comparison between the circulating and salivary levels of these three cytokines has been widely explored,^122–125^ showing overall modest reliability of saliva and low consistency. The only one marker that may more precisely reflects blood distribution is IL-6.^126,127^ Indeed, a significant correlation between plasma and saliva has been reported in patients affected by IBD and oral lichen planus (OLP).^56,126^ Furthermore, IL-8 and MMP-8 were found to be increased in patients with head and neck squamous cell cancer or bowel diseases and in those suffering of diabetes or who underwent cardiac surgery, respectively.^128^ In detail, a population study across 441 adults described that diabetic patients have a three times higher ratio of MMP-8/TIMP-1 and twice as high concentration of MMP-8, as a consequence of the elevated inflammatory *status* in these patients.^128^ According to this notion, MMP-8 has been found to be up-regulated in patients with elevated risk of CVD.^128,129^

Among salivary biomarkers, miRNAs seem very promising, both for the early diagnosis and for understanding the pathogenesis of some diseases (e.g., oral cancer, salivary glands cancer, neurological or psychiatric deficiencies).^130^ Moreover, it has been demonstrated that salivary transcriptome is very abundant, consisting of thousands of mRNAs and miRNAs.^131,132^

In saliva samples of patients with oral squamous cell carcinoma (OSCC), miRNA-125a and miR-200a were significantly decreased and miR-31 was over-expressed. Aberrant methylation and atypical expression were observed for miR-200c/miR-141 and miR-375/miR-200a, respectively.^133^ In addition, promising biomarkers were represented by miR-768-3p and miR-574 for salivary gland inflammation and by miR-5100 for Sjögren’s syndrome.^134,135^ Other miRNAs whose expression is deregulated in saliva include miR-101 in Crohn’s disease and miR-21, miR-31, miR-142-3p/5p in ulcerative colitis.^136^ Evidence exists that salivary miR-940 and miR-3679-5p are reliable markers for pancreatic cancer whereas miR140-5p and miR301a are attractive molecules for the salivary diagnosis of gastric cancer.^137^

In addition to the more traditional inflammatory biomarkers, salivary levels of lipid mediators can also be used for diagnostic and prognostic purposes despite their investigation has been given little scientific attention and remains poorly understood.^138^ A study of serum and saliva lipid profile levels in about 100 healthy subjects showed that there is a reasonable correlation between their concentration of total cholesterol and triglycerides.^139^ The measurement of short-chain fatty acids (SCFAs) in saliva, produced by causal bacteria, may be an indicator of the inflammation degree closely related to the onset and progression of periodontal disease.^140,141^ Elevated salivary leukotriene B_4_ (LTB_4_) and PGE_2_ are correlated with arterial stiffness.^142^ Asthmatic patients exhibit elevated Cys-leukotriens (LTs) levels in saliva.^143^ Elevated salivary levels of PGE_2_ were found to be correlated with gingivitis^144,145^ and periodontitis.^146^ In chronic periodontitis patients, salivary LTB_4_ levels were correlated with the severity of alveolar bone loss.^147^ Regarding the primary Sjögren’s syndrome (SS), Slomiany et al. demonstrated a general increase in total salivary lipid count in SS patients as well as an increased proportion of glycolipids, phospholipids and some neutral lipids.^148^ Another study analyzing eicosanoids reported an increase in PGE_2_ and thromboxane B_2_ (TXB_2_) in SS patients when compared to healthy controls.^149^ Fineide et al. revealed several significant differences in the lipidomic profiles of saliva in human patients suffering from SS compared to healthy controls showing increasing levels of sphingomyelins and diacylglycerophosphocholines and decreasing levels of diacylglycerols and ceramides in unstimulated saliva from SS patients.^150^

### Salivary biomarkers of oxidative stress

Persistent inflammation and blunted antioxidant capacities resulted in the exaggerate generation of free radicals which propagate injuries, precipitating cell death. The release of these harmful radicals, along with reactive oxygen and nitrogen species (ROS/RNS) boost the activation of signaling molecules and transcription factors, that may be particularly useful to pinpoint the disorders. In detail, free radicals are responsible for the oxidation of cellular components, such as membrane lipids, proteins, and nucleic acids, contributing to mitochondrial dysfunction, antioxidant systems impairment, ageing and chronic diseases.^151–153^ Products derived from lipid peroxidation, protein oxidation and DNA damage can be directly assessed in saliva, possibly paving the way to diagnose systemic disorders associated with oxidative stress, by using this mean.^152,154,155^ Nonetheless, local oral status and oral cavity-related pathologies (i.e., periodontitis and dental caries) may also modulate the redox balance of saliva, interfering with its widespread routine clinical use.^156^

A broad number of studies indicate that an imbalance in oxidant/antioxidant mediators may exert a crucial role in the pathogenesis and progression of metabolic syndrome, T2D and CVD.^157^ However, the majority of the research is focused on tissue and blood distribution of these indicators, and less is known regarding their impact on saliva composition. A preclinical study in insulin resistant rats compared salivary antioxidants and oxidative stress products to those in plasma samples, exploring their diagnostic utility. The authors showed an impairment in antioxidant barriers and in ROS scavengers both in plasma and in saliva, proven by the reduction in superoxide dismutase, ascorbic acid and glutathione (GSH) levels in IR mice.^158^ These alterations were paralleled by strengthened lipid/protein oxidation and advanced glycation end products in both biological fluids, showing an elevated coefficient of correlation between the two.

The assessment of salivary redox biomarkers seems to be applicable also for diagnosis and monitoring of obesity,^159,160^ diabetes,^161^ hypertensive disorders,^162^ chronic kidney disease,^163^ heart failure,^164,165^ neurodegenerative diseases^166^ and cancer,^167,168^ in which molecules and enzymes with antioxidant properties are pathologically depleted in saliva, whereas oxidative and nitrosative by-products are favored. For instance, it has been demonstrated that salivary oxidative biomarkers, among which 4-hydroxynonenal (4-HNE) and 8-isoprostanes (8-isoP), advanced oxidation protein products (AOPP) and protein carbonyl groups (PC), 8-hydroxy-D-guanosine (8-OHdG), derived from lipoperoxidation of cell membranes, protein oxidation and DNA aberrancies respectively, were increased in 47 subjects with morbid obesity compared to controls (BMI < 25 kg·m^−2^) and that bariatric surgery reduced their salivary concentrations.^160^ Similar findings have been reported by Zalewska and colleagues, which yielded an enhanced total oxidative status accompanied by reduced glutathione levels in saliva and in plasma from 40 young obese subjects.^159^ Other important markers of oxidative damage are mitochondrial DNA mutations, which has been evaluated in blood and gingival tissues.^153^

Higher levels of glutathione peroxidase (GSHPx), an antioxidant enzyme, and malondialdehyde (MDA), a biomarker for lipid peroxidation, were observed in the saliva of patients that had periodontitis and that were smokers compared to the non-smoking control group.^169,170^ Therefore, increased levels of GSHPx and MDA can indicate increased lipid peroxidation in patients with periodontal disease which is further elevated by smoking.^169,170^ The results of Wolfram et al.^171^ and Morrow et al.^172^ indicate that salivary lipid isoprostanes (IPs) can reliably assess the degree of oxidative stress. In detail, elevated salivary 8-iso-PGF_2α_ levels were determined by oxidative damage associated with the extent of periodontal disease and significantly aggravated by concomitant tobacco use. Likewise, the levels of salivary PGE_2_, PGF_2α_ and prostaglandin D_2_ (PGD_2_) have been successfully used as biomarkers for chronic inflammatory processes and to assess the degree of oxidative stress caused by smoking and periodontitis. The results of Huang et al. demonstrate that a local redox alteration contributes significantly to periodontitis through the modulation of fatty acid metabolism in response to inflammation and oxidative stress.^173^

In addition, smoking induces specific structural alterations in the lipid A-derived 3-hydroxy (OH) fatty acid profile in saliva of individuals with chronic periodontitis that are consistent with an altered oral microflora.^174^ The most prominent shifts in smokers, compared to non-smokers, occurred in the short, straight-chain pro-inflammatory lipid A fatty acids, 3-OH C12, C13, and C14.

Furthermore, the concentration of salivary redox biomarkers progressively increases according to the disease progression, mirroring their presence at the serum level.^175,176^ For instance, the salivary content of 8-OHdG, MDA, and PC was significantly higher in patients affected by CAD compared to healthy individuals, supporting the paramount role of lipid oxidative damage in the etiology of CVD. Even more, in these subjects, MDA levels were associated with serum high sensitivity CRP (hsCRP) and with plasma fibrinogen, that are strong predictors of cardiovascular events.^176^

## Conclusions

The measurement of soluble mediators outlines the entire therapeutic route from diagnosis to therapy and follow-up. In detail, a large series of acute phase proteins, cytokines and chemokines, lipids, pro-inflammatory enzymes, and oxidative stress indicators have been pointed out as trustworthy biomarkers in biological fluids. Since chronic disorders, among which cardiovascular failure, obesity, diabetes, and cancer, have been progressively spread in the last century, there is an urgent need to identify novel diagnostic strategies to tailor the management of patients and to stage the diseases. In this regard, ever increasing number of studies have suggested that salivary biomarkers modulation will be an innovative and minimally invasive option in the care of chronically affected patients. Hence, addressing the efforts to the research of peculiar salivary molecules will provide clinicians an unprecedented opportunity to soften the painful path of the disease.

Furthermore, technological advances enable the implementation of wearable biosensors in the oral cavity that automates the detection and quantification of biomarkers in saliva. That allows early, non-invasive and unobtrusive diagnosis, continuous monitoring of chronic disease and early and continuous communication with physicians and thus improve the quality of patient care while reducing the cost of care.

**Fig. 5:**
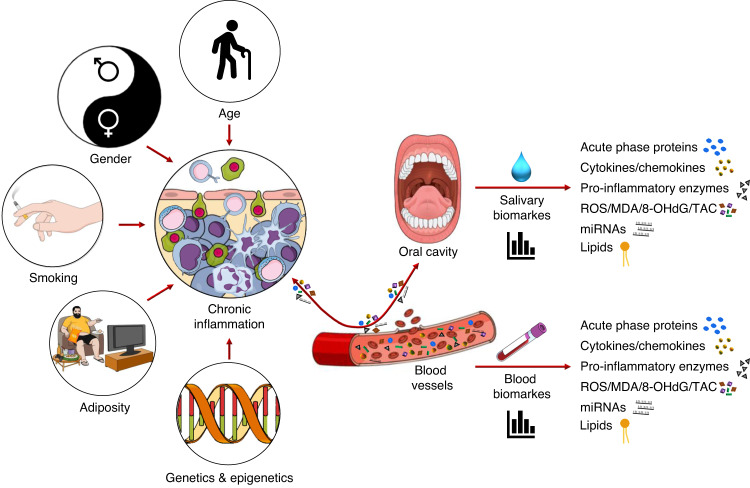
Schematic illustration of possible modifiers of chronic inflammation, analytes enchanged between blood and oral cavity, and dosable compounds in biological fluids

The possibility to assign a specific spectrum of candidate molecules and detecting salivary technologies to discriminate each stage of chronic disorders and to formulate panels of salivary mediators as suitable molecular biomarkers to be combined with the demographic, genetic and anthropometric features of patients, might represent a novel tool to improve the diagnosis and more accurately evaluate the prognosis (Fig. 5).

## References

[1] Furman D (2019). Chronic inflammation in the etiology of disease across the life span. Nat. Med..

[2] Calder PC (2013). A consideration of biomarkers to be used for evaluation of inflammation in human nutritional studies. Br. J. Nutr..

[3] Raghupathi W, Raghupathi V (2018). An Empirical Study of Chronic Diseases in the United States: A Visual Analytics Approach. Int. J. Environ. Res. Public Health.

[4] Prasad S, Tyagi AK, Aggarwal BB (2016). Detection of inflammatory biomarkers in saliva and urine: potential in diagnosis, prevention, and treatment for chronic diseases. Exp. Biol. Med..

[5] Edgar WM (1992). Saliva: its secretion, composition and functions. Br. Dent. J..

[6] Pfaffe T (2011). Diagnostic potential of saliva: current state and future applications. Clin. Chem..

[7] Karjalainen S (1997). Salivary cholesterol of healthy adults in relation to serum cholesterol concentration and oral health. J. Dent. Res..

[8] D RM (2014). Evaluation of salivary flow rate, pH and buffer in pre, post & post menopausal women on HRT. J. Clin. Diagn. Res..

[9] Inoue H (2006). Gender difference in unstimulated whole saliva flow rate and salivary gland sizes. Arch. Oral. Biol..

[10] Srivastava A (2008). Age and gender related differences in human parotid gland gene expression. Arch. Oral. Biol..

[11] Prodan A (2015). Interindividual variation, correlations, and sex-related differences in the salivary biochemistry of young healthy adults. Eur. J. Oral. Sci..

[12] Schepici G, Silvestro S, Trubiani O, Bramanti P, Mazzon E (2020). Salivary biomarkers: future approaches for early diagnosis of neurodegenerative diseases. Brain Sci..

[13] Pawlik P, Blochowiak K (2021). The role of salivary biomarkers in the early diagnosis of Alzheimer’s disease and Parkinson’s disease. Diagnostics (Basel).

[14] Ershler WB (1993). Interleukin-6: a cytokine for gerontologists. J. Am. Geriatr. Soc..

[15] Ferrucci L (2005). The origins of age-related proinflammatory state. Blood.

[16] Brüünsgaard H, Pedersen BK (2003). Age-related inflammatory cytokines and disease. Immunol. Allergy Clin. North Am..

[17] Ridker PM (2000). Plasma concentration of interleukin-6 and the risk of future myocardial infarction among apparently healthy men. Circulation.

[18] Dinarello CA (2006). Interleukin 1 and interleukin 18 as mediators of inflammation and the aging process. Am. J. Clin. Nutr..

[19] Rea IM (1996). Changes in lymphocyte subsets, interleukin 2, and soluble interleukin 2 receptor in old and very old age. Gerontology.

[20] Rübenhagen R (2012). Interleukin-7 levels in synovial fluid increase with age and MMP-1 levels decrease with progression of osteoarthritis. Acta Orthop..

[21] Zykov MV (2016). Interleukin-12 serum level has prognostic value in patients with ST-segment elevation myocardial infarction. Heart Lung.

[22] Garrett-Sinha LA, John S, Gaffen SL (2008). IL-17 and the Th17 lineage in systemic lupus erythematosus. Curr. Opin. Rheumatol..

[23] Rink L, Cakman I, Kirchner H (1998). Altered cytokine production in the elderly. Mech. Ageing Dev..

[24] Greenfield JR, Campbell LV (2006). Relationship between inflammation, insulin resistance and type 2 diabetes: ‘cause or effect’?. Curr. Diabetes Rev..

[25] Visser M (1999). Elevated C-reactive protein levels in overweight and obese adults. Jama.

[26] Corica F (1999). Relationship between plasma leptin levels and the tumor necrosis factor-alpha system in obese subjects. Int. J. Obes. Relat. Metab. Disord..

[27] Fantuzzi G (2005). Adipose tissue, adipokines, and inflammation. J. Allergy Clin. Immunol..

[28] Raitakari M (2005). Distribution and determinants of serum high-sensitive C-reactive protein in a population of young adults: the Cardiovascular Risk in Young Finns Study. J. Intern. Med..

[29] Marques-Vidal P (2011). Levels and determinants of inflammatory biomarkers in a Swiss population-based sample (CoLaus study). PLoS ONE.

[30] Bouman A (2004). Gender difference in the non-specific and specific immune response in humans. Am. J. Reprod. Immunol..

[31] Tessaro FH, Ayala TS, Martins JO (2015). Lipid mediators are critical in resolving inflammation: a review of the emerging roles of eicosanoids in diabetes mellitus. Biomed. Res. Int..

[32] Balgoma D (2016). Linoleic acid-derived lipid mediators increase in a female-dominated subphenotype of COPD. Eur. Respir. J..

[33] Pomponi MF (2011). Why docosahexaenoic acid and aspirin supplementation could be useful in women as a primary prevention therapy against Alzheimer’s disease?. Ageing Res. Rev..

[34] Yu D (2015). Inverse relationship between serum lipoxin A4 level and the risk of metabolic syndrome in a middle-aged Chinese population. PLoS ONE.

[35] Lee J, Taneja V, Vassallo R (2012). Cigarette smoking and inflammation: cellular and molecular mechanisms. J. Dent. Res..

[36] Elisia I (2020). The effect of smoking on chronic inflammation, immune function and blood cell composition. Sci. Rep..

[37] Strzelak A, Ratajczak A, Adamiec A, Feleszko W (2018). Tobacco smoke induces and alters immune responses in the lung triggering inflammation, allergy, asthma and other lung diseases: a mechanistic review. Int. J. Environ. Res. Public Health..

[38] Van Dyke TE (2007). Control of inflammation and periodontitis. Periodontol 2000.

[39] Delanty N (1996). 8-Epi PGF2 alpha: specific analysis of an isoeicosanoid as an index of oxidant stress in vivo. Br. J. Clin. Pharm..

[40] Reilly M (1996). Modulation of oxidant stress in vivo in chronic cigarette smokers. Circulation.

[41] Alpagot T (2007). Longitudinal evaluation of prostaglandin E2 (PGE2) and periodontal status in HIV+ patients. Arch. Oral. Biol..

[42] Ng PY (2007). Candidate salivary biomarkers associated with alveolar bone loss: cross-sectional and in vitro studies. FEMS Immunol. Med. Microbiol..

[43] Shanmugam MK, Sethi G (2013). Role of epigenetics in inflammation-associated diseases. Subcell. Biochem..

[44] Gonzalez-Jaramillo V (2019). Epigenetics and inflammatory markers: a systematic review of the current evidence. Int. J. Inflam..

[45] Marques-Rocha JL (2015). Noncoding RNAs, cytokines, and inflammation-related diseases. FASEB J..

[46] Prats-Puig A (2013). Changes in circulating microRNAs are associated with childhood obesity. J. Clin. Endocrinol. Metab..

[47] Sun Y (2020). Inhibition of miR-153, an IL-1β-responsive miRNA, prevents beta cell failure and inflammation-associated diabetes. Metabolism.

[48] Jiang F (2020). Hepatocyte-derived extracellular vesicles promote endothelial inflammation and atherogenesis via microRNA-1. J. Hepatol..

[49] Nunomura A, Perry G (2020). RNA and oxidative stress in Alzheimer’s disease: focus on microRNAs. Oxid. Med. Cell Longev..

[50] Markopoulos GS (2018). Roles of NF-κB Signaling in the Regulation of miRNAs Impacting on Inflammation in Cancer. Biomedicines.

[51] Capurso C (2004). Interleukin 6-174 G/C promoter gene polymorphism and sporadic Alzheimer’s disease: geographic allele and genotype variations in Europe. Exp. Gerontol..

[52] Humphries SE (2001). The interleukin-6 -174 G/C promoter polymorphism is associated with risk of coronary heart disease and systolic blood pressure in healthy men. Eur. Heart J..

[53] Elahi MM (2009). Tumor necrosis factor alpha -308 gene locus promoter polymorphism: an analysis of association with health and disease. Biochim. Biophys. Acta.

[54] Lio D (2003). Inflammation, genetics, and longevity: further studies on the protective effects in men of IL-10 -1082 promoter SNP and its interaction with TNF-alpha -308 promoter SNP. J. Med. Genet..

[55] Wilson, S. J., Woody, A. & Kiecolt-Glaser, J. K. *Inflammation As a Biomarker Method in Lifespan Developmental Methodology* (Oxford University Press, 2018).

[56] Williamson S (2012). Comparison of biomarkers in blood and saliva in healthy adults. Nurs. Res. Pr..

[57] Kumar S, Padmashree S, Jayalekshmi R (2014). Correlation of salivary glucose, blood glucose and oral candidal carriage in the saliva of type 2 diabetics: a case-control study. Contemp. Clin. Dent..

[58] Adebero T (2020). Salivary and serum concentrations of cortisol and testosterone at rest and in response to intense exercise in boys versus men. Pediatr. Exerc. Sci..

[59] Nam Y (2019). Salivary biomarkers of inflammation and oxidative stress in healthy adults. Arch. Oral. Biol..

[60] Granger DA (2012). Incorporating salivary biomarkers into nursing research: an overview and review of best practices. Biol. Res. Nurs..

[61] Padilla, G. A. et al. Saliva Collection, Handling, Transport, and Storage: Special Considerations and Best Practices for Interdisciplinary Salivary Bioscience Research, in Salivary Bioscience: Foundations of Interdisciplinary Saliva Research and Applications (eds Granger, D. A. & Taylor, M. K.). 21–47 (Springer International Publishing, Cham, 2020).

[62] Orive G, Lopera F, Carro E (2022). Saliva is a good candidate to be the new gold-standard sample for neurodegenerative diseases. J. Alzheimers Dis..

[63] Lakshmi K (2017). Oral fluid-based biosensors: a novel method for rapid and noninvasive diagnosis. Indian J. Dent. Sci..

[64] Eftekhari A (2019). Bioassay of saliva proteins: the best alternative for conventional methods in non-invasive diagnosis of cancer. Int. J. Biol. Macromol..

[65] García-Carmona, L. et al. Pacifier Biosensor: toward noninvasive saliva biomarker monitoring. *Anal. Chem*. **91**, 13883–13891 (2019). 10.1021/acs.analchem.9b03379 31573188

[66] Goldoni R (2022). Salivary biomarkers of neurodegenerative and demyelinating diseases and biosensors for their detection. Ageing Res. Rev..

[67] Simón-Soro A (2013). Microbial geography of the oral cavity. J. Dent. Res..

[68] Gug IT (2019). Salivary biomarkers detection: analytical and immunological methods overview. TrAC Trends Anal. Chem..

[69] Mohamed R (2012). The impact of saliva collection and processing methods on CRP, IgE, and Myoglobin immunoassays. Clin. Transl. Med..

[70] Pappa, E. & Kousvelari E. Saliva in the “Omics” era: a promising tool in paediatrics. *Oral Dis*. **25**, 16–25 (2019). 10.1111/odi.12886 29750386

[71] Shirtcliff EA (2001). Use of salivary biomarkers in biobehavioral research: cotton-based sample collection methods can interfere with salivary immunoassay results. Psychoneuroendocrinology.

[72] Minetto MA (2007). Influence of the sample collection method on salivary interleukin–6 levels in resting and post-exercise conditions. Eur. J. Appl. Physiol..

[73] Goldoni R (2021). Recent advances in graphene-based nanobiosensors for salivary biomarker detection. Biosens. Bioelectron..

[74] Goldoni R (2021). Malignancies and Biosensors: A Focus on Oral Cancer Detection through Salivary Biomarkers. Biosensors (Basel).

[75] Herr AE (2007). Microfluidic immunoassays as rapid saliva-based clinical diagnostics. Proc. Natl Acad. Sci. USA.

[76] Yee EH (2017). Detection of biomarkers of periodontal disease in human saliva using stabilized, vertical flow immunoassays. ACS Sens..

[77] Jung DG, Jung D, Kong SH (2017). A lab-on-a-chip-based non-invasive optical sensor for measuring glucose in saliva. Sensors.

[78] Rossini EL (2021). Paper microfluidic device using carbon dots to detect glucose and lactate in saliva samples. Spectrochim. Acta Part A: Mol. Biomol. Spectrosc..

[79] Helton KL (2008). Conditioning saliva for use in a microfluidic biosensor. Lab Chip.

[80] Herr AE (2007). Microfluidic immunoassays as rapid saliva-based clinical diagnostics. Proc. Natl Acad. Sci. USA.

[81] Lee YJ (2020). Optimization of Saliva Collection and Immunochromatographic Detection of Salivary Pepsin for Point-of-Care Testing of Laryngopharyngeal Reflux. Sensors (Basel).

[82] Malon RSP (2014). Saliva-based biosensors: noninvasive monitoring tool for clinical diagnostics. BioMed. Res. Int..

[83] Lukose, J. et al. Photonics of human saliva: potential optical methods for the screening of abnormal health conditions and infections. *Biophys. Rev*. **13**, 359–385 (2021). 10.1007/s12551-021-00807-8 PMC8170462 34093888

[84] Suni II (2021). Substrate materials for biomolecular immobilization within electrochemical biosensors. Biosensors (Basel).

[85] Liang X (2021). Carbon-based SERS biosensor: from substrate design to sensing and bioapplication. NPG Asia Mater..

[86] Kim J (2014). Non-invasive mouthguard biosensor for continuous salivary monitoring of metabolites. Analyst.

[87] Chen, Y., Xianyu, Y. & Jiang, X. Surface modification of gold nanoparticles with small molecules for biochemical analysis. *Acc. Chem. Res*. **50**, 310–319 (2017). 10.1021/acs.accounts.6b00506 28068053

[88] Guo Q, Li F (2014). Self-assembled alkanethiol monolayers on gold surfaces: resolving the complex structure at the interface by STM. Phys. Chem. Chem. Phys..

[89] Zamani, M. et al. Surface requirements for optimal biosensing with disposable gold electrodes. *ACS Meas. Sci. Au*. **2**, 91–95 (2022) 10.1021/acsmeasuresciau.1c00042 PMC9026247 35479101

[90] Samanta D, Sarkar A (2011). Immobilization of bio-macromolecules on self-assembled monolayers: methods and sensor applications. Chem. Soc. Rev..

[91] Mani V (2021). Electrochemical sensors targeting salivary biomarkers: a comprehensive review. TrAC Trends Anal. Chem..

[92] Lukose J (2021). Photonics of human saliva: potential optical methods for the screening of abnormal health conditions and infections. Biophys. Rev..

[93] Taylor JJ (2019). A prototype antibody-based biosensor for measurement of salivary MMP-8 in periodontitis using surface acoustic wave technology. Sci. Rep..

[94] Huang Y, Das PK, Bhethanabotla VR (2021). Surface acoustic waves in biosensing applications. Sens. Actuators Rep..

[95] BelBruno JJ (2019). Molecularly imprinted polymers. Chem. Rev..

[96] Fedorenko V (2021). Application of polydopamine functionalized zinc oxide for glucose biosensor design. Polymers (Basel).

[97] Kim J (2015). Wearable salivary uric acid mouthguard biosensor with integrated wireless electronics. Biosens. Bioelectron..

[98] Lee, Y. et al. Wireless, intraoral hybrid electronics for real-time quantification of sodium intake toward hypertension management. *Proc. Natl Acad. Sci. USA* **115**, 5377–5382 (2018). 10.1073/pnas.1719573115 PMC6003521 29735689

[99] Mannoor MS (2012). Graphene-based wireless bacteria detection on tooth enamel. Nat. Commun..

[100] Tseng P (2018). Functional, RF-trilayer sensors for tooth-mounted, wireless monitoring of the oral cavity and food consumption. Adv. Mater..

[101] Ludwig N (2019). Machine learning to detect Alzheimer’s disease from circulating non-coding RNAs. Genomics Proteom. Bioinforma..

[102] Li Z (2022). Identifying key microRNA signatures for neurodegenerative diseases with machine learning methods. Front. Genet..

[103] Ko J (2018). Machine learning to detect signatures of disease in liquid biopsies – a user’s guide. Lab a Chip.

[104] Khamina K (2022). A microRNA next-generation-sequencing discovery assay (miND) for genome-scale analysis and absolute quantitation of circulating microRNA biomarkers. Int. J. Mol. Sci..

[105] Sander LE (2010). Hepatic acute-phase proteins control innate immune responses during infection by promoting myeloid-derived suppressor cell function. J. Exp. Med..

[106] Jain S, Gautam V, Naseem S (2011). Acute-phase proteins: as diagnostic tool. J. Pharm. Bioallied. Sci..

[107] Byrne ML (2013). Acute phase protein and cytokine levels in serum and saliva: a comparison of detectable levels and correlations in a depressed and healthy adolescent sample. Brain Behav. Immun..

[108] Gulhar, R., Ashraf, M. A. & Jialal, I. Physiology, acute phase reactants, in StatPearls. StatPearls Publishing Copyright © 2022, (StatPearls Publishing LLC., Treasure Island (FL), 2022). 30137854

[109] Devaraj S, Singh U, Jialal I (2009). The evolving role of C-reactive protein in atherothrombosis. Clin. Chem..

[110] Megson E (2010). C-reactive protein in gingival crevicular fluid may be indicative of systemic inflammation. J. Clin. Periodontol..

[111] Pay JB, Shaw AM (2019). Towards salivary C-reactive protein as a viable biomarker of systemic inflammation. Clin. Biochem..

[112] Ouellet-Morin I (2011). Validation of a high-sensitivity assay for C-reactive protein in human saliva. Brain Behav. Immun..

[113] Cho, Y. R. & Oh, Y. I. Comparative analysis of C-reactive protein levels in the saliva and serum of dogs with various diseases. *Animals* **10**, 1042 (2020). 10.3390/ani10061042 PMC7341185 32560466

[114] Szabo YZ, Slavish DC (2021). Measuring salivary markers of inflammation in health research: a review of methodological considerations and best practices. Psychoneuroendocrinology.

[115] Jacobs R (2016). Host biomarkers detected in saliva show promise as markers for the diagnosis of pulmonary tuberculosis disease and monitoring of the response to tuberculosis treatment. Cytokine.

[116] Helmi ZR, Sabri RA, Hameed BH (2016). Assessment of oral health status, leptin, and inflammatory markers in serum and saliva of patients with polycystic ovarian syndrome in reference to metabolic syndrome. Mustansiriya Med. J..

[117] Sali V (2021). Dynamics of salivary adenosine deaminase, haptoglobin, and cortisol in lipopolysaccharide-challenged growing pigs. Front. Vet. Sci..

[118] Bozovic D, Racic M, Ivkovic N (2013). Salivary cortisol levels as a biological marker of stress reaction. Med. Arch..

[119] Arafah BM (2007). Measurement of salivary cortisol concentration in the assessment of adrenal function in critically ill subjects: a surrogate marker of the circulating free cortisol. J. Clin. Endocrinol. Metab..

[120] Palmier, N. R. & Leme, A. F. P. Salivary alpha-1-antitrypsin and macrophage migration inhibitory factor may be potential prognostic biomarkers for oncologic treatment-induced severe oral mucositis. *Support Care Cancer* **29**, 2939–2946 (2021). 10.1007/s00520-020-05805-2 33009579

[121] Slavish DC (2015). Salivary markers of inflammation in response to acute stress. Brain Behav. Immun..

[122] Fernandez-Botran R (2011). Correlations among inflammatory markers in plasma, saliva and oral mucosal transudate in post-menopausal women with past intimate partner violence. Brain Behav. Immun..

[123] Izawa S (2013). The diurnal patterns of salivary interleukin-6 and C-reactive protein in healthy young adults. Brain Behav. Immun..

[124] Riis JL (2014). Salivary cytokines in healthy adolescent girls: Intercorrelations, stability, and associations with serum cytokines, age, and pubertal stage. Dev. Psychobiol..

[125] Riis JL (2015). Salivary cytokines as a minimally-invasive measure of immune functioning in young children: correlates of individual differences and sensitivity to laboratory stress. Dev. Psychobiol..

[126] Aleksandra Nielsen A (2005). Saliva Interleukin-6 in patients with inflammatory bowel disease. Scand. J. Gastroenterol..

[127] Äyräväinen, L. et al. Inflammatory biomarkers in saliva and serum of patients with rheumatoid arthritis with respect to periodontal status. *Ann. Med*. **50**, 333–344 (2018). 10.1080/07853890.2018.1468922 29683364

[128] Rathnayake N (2013). Salivary biomarkers for detection of systemic diseases. PLoS ONE.

[129] Kosaka T (2014). Salivary inflammatory cytokines may be novel markers of carotid atherosclerosis in a Japanese general population: the Suita study. Atherosclerosis.

[130] Rapado-González Ó (2018). Human salivary microRNAs in Cancer. J. Cancer.

[131] Park NJ (2009). Salivary microRNA: discovery, characterization, and clinical utility for oral cancer detection. Clin. Cancer Res..

[132] Michael A (2010). Exosomes from human saliva as a source of microRNA biomarkers. Oral. Dis..

[133] Dang J (2013). MicroRNA-137 promoter methylation in oral lichen planus and oral squamous cell carcinoma. J. Oral. Pathol. Med..

[134] Jiang J (2005). Real-time expression profiling of microRNA precursors in human cancer cell lines. Nucleic Acids Res..

[135] Tandon M (2012). Deep sequencing of short RNAs reveals novel microRNAs in minor salivary glands of patients with Sjögren’s syndrome. Oral. Dis..

[136] Nijakowski K, Surdacka A (2020). Salivary biomarkers for diagnosis of inflammatory bowel diseases: a systematic review. Int. J. Mol. Sci..

[137] Setti G (2020). Salivary microRNA for diagnosis of cancer and systemic diseases: a systematic review. Int. J. Mol. Sci..

[138] Sommakia S, Baker OJ (2017). Regulation of inflammation by lipid mediators in oral diseases. Oral. Dis..

[139] Singh S (2014). Evaluation of serum and salivary lipid profile: a correlative study. J. Oral. Maxillofac. Pathol..

[140] Hatanaka, K. et al. Enzymatic measurement of short-chain fatty acids and application in periodontal disease diagnosis. *PLoS ONE* **17**, e0268671 (2022). 10.1371/journal.pone.0268671 PMC9286277 35839206

[141] Kawase T (2020). Simultaneous determination of 7 short-chain fatty acids in human saliva by high-sensitivity gas chromatography-mass spectrometry. CHROMATOGRAPHY.

[142] Labat C (2013). Inflammatory mediators in saliva associated with arterial stiffness and subclinical atherosclerosis. J. Hypertens..

[143] Gaber F (2008). Increased levels of cysteinyl-leukotrienes in saliva, induced sputum, urine and blood from patients with aspirin-intolerant asthma. Thorax.

[144] Syndergaard B (2014). Salivary biomarkers associated with gingivitis and response to therapy. J. Periodontol..

[145] Gümüş P (2016). Evaluation of the gingival inflammation in pregnancy and postpartum via 25-hydroxy-vitamin D3, prostaglandin E2 and TNF-α levels in saliva. Arch. Oral. Biol..

[146] Sánchez GA (2013). Salivary IL-1β and PGE2 as biomarkers of periodontal status, before and after periodontal treatment. J. Clin. Periodontol..

[147] Sánchez GA (2013). Relationship between salivary leukotriene B4 levels and salivary mucin or alveolar bone resorption, in subjects with periodontal health and disease. J. Periodontal Res..

[148] Slomiany BL (1986). Lipid composition and viscosity of parotid saliva in Sjögren syndrome in man. Arch. Oral. Biol..

[149] Tishler M (1996). Salivary eicosanoid concentration in patients with Sjögren’s syndrome. Ann. Rheum. Dis..

[150] Fineide F (2021). Characterization of Lipids in saliva, tears and minor salivary glands of Sjögren’s Syndrome patients using an HPLC/MS-based approach. Int. J. Mol. Sci.

[151] Longo M (2022). TM6SF2/PNPLA3/MBOAT7 loss-of-function genetic variants impact on NAFLD development and progression both in patients and in in vitro models. Cell Mol. Gastroenterol. Hepatol..

[152] Maciejczyk M, Zalewska A, Gerreth AK (2020). Salivary redox biomarkers in selected neurodegenerative diseases. J. Clin. Med.

[153] Bullon P, Newman HN, Battino M (2014). Obesity, diabetes mellitus, atherosclerosis and chronic periodontitis: a shared pathology via oxidative stress and mitochondrial dysfunction?. Periodontol 2000.

[154] Maciejczyk, M. & Nesterowicz, M. Oxidation, glycation, and carbamylation of salivary biomolecules in healthy children, adults, and the elderly: can saliva be used in the assessment of aging? *J. Inflamm. Res*. **15**, 2051–2073 (2022). 10.2147/JIR.S356029 PMC8976116 35378954

[155] Šteňová, E., Bakošová M. & Lauková, L. Biological anti-TNF-α therapy and markers of oxidative and carbonyl stress in patients with rheumatoid arthritis. *Oxid. Med. Cell Longev*. **2021**, 5575479 (2021). 10.1155/2021/5575479 PMC8716244 34976302

[156] Tóthová L (2015). Salivary markers of oxidative stress in oral diseases. Front. Cell Infect. Microbiol.

[157] Vona, R. & Gambardella, L. Biomarkers of oxidative stress in metabolic syndrome and associated diseases. *Oxid. Med. Cell Longev*. **2019**, 8267234 (2019). 10.1155/2019/8267234 PMC6525823 31191805

[158] Maciejczyk, M. et al. Salivary redox biomarkers in insulin resistance: preclinical studies in an animal model. *Oxid. Med. Cell Longev*. **2021**, 3734252 (2021). 10.1155/2021/3734252 PMC8455206 34557264

[159] Zalewska A (2020). Dysfunction of salivary glands, disturbances in salivary antioxidants and increased oxidative damage in saliva of overweight and obese adolescents. J. Clin. Med.

[160] Fejfer K (2017). Oxidative modification of biomolecules in the nonstimulated and stimulated saliva of patients with morbid obesity treated with bariatric surgery. Biomed. Res. Int..

[161] Smriti K (2016). Salivary glucose as a diagnostic marker for diabetes mellitus. J. Diabetes Sci. Technol..

[162] Maciejczyk M, Taranta-Janusz K, Wasilewska A, Kossakowska A, Zalewska A (2020). A case-control study of salivary redox homeostasis in hypertensive children. Can Salivary Uric Acid be a Marker of Hypertension?. J. Clin. Med..

[163] Maciejczyk M, Szulimowska J, Taranta-Janusz K, Wasilewska A, Zalewska A (2020). Salivary gland dysfunction, protein glycooxidation and nitrosative stress in children with chronic kidney disease. J. Clin. Med..

[164] Ghimenti, S. & Lomonaco, T. Salivary lactate and 8-isoprostaglandin F(2α) as potential non-invasive biomarkers for monitoring heart failure: a pilot study. *Sci. Rep*. **10**, 7441 (2020) 10.1038/s41598-020-64112-2 PMC7198483 32366899

[165] Abdul Rehman S (2017). Role of salivary biomarkers in detection of cardiovascular diseases (CVD). Proteomes.

[166] Galindez JM (2021). Salivary heme oxygenase-1: a potential biomarker for central neurodegeneration. J. Cent. Nerv. Syst. Dis..

[167] Gornitsky M (2016). Altered levels of salivary 8-oxo-7-hydrodeoxyguanosine in breast cancer. JDR Clin. Trans. Res..

[168] Porto-Mascarenhas EC (2017). Salivary biomarkers in the diagnosis of breast cancer: a review. Crit. Rev. Oncol. Hematol..

[169] Guentsch A (2008). Lipid peroxidation and antioxidant activity in saliva of periodontitis patients: effect of smoking and periodontal treatment. Clin. Oral. Investig..

[170] Wenk MR (2005). The emerging field of lipidomics. Nat. Rev. Drug Discov..

[171] Wolfram RM (2006). Salivary isoprostanes indicate increased oxidation injury in periodontitis with additional tobacco abuse. Biofactors.

[172] Morrow JD (1995). Increase in circulating products of lipid peroxidation (F2-isoprostanes) in smokers. Smoking as a cause of oxidative damage. N. Engl. J. Med..

[173] Huang Y (2014). Mass spectrometry-based metabolomic profiling identifies alterations in salivary redox status and fatty acid metabolism in response to inflammation and oxidative stress in periodontal disease. Free Radic. Biol. Med..

[174] Buduneli N (2011). Fatty acid profiles in smokers with chronic periodontitis. J. Dent. Res..

[175] Kułak-Bejda A, Waszkiewicz N, Bejda G, Zalewska A, Maciejczyk M (2019). Diagnostic value of salivary markers in neuropsychiatric disorders. Disease Markers.

[176] Nguyen TT (2017). Salivary oxidative stress biomarkers in chronic periodontitis and acute coronary syndrome. Clin. Oral. Investig..

[177] Ciui B (2019). Cavitas electrochemical sensor toward detection of N-epsilon (carboxymethyl)lysine in oral cavity. Sens. Actuators B: Chem..

[178] Arakawa, T. et al. A wearable cellulose acetate-coated mouthguard biosensor for in vivo salivary glucose measurement. *Anal. Chem*. **92**, 12201–12207 (2020). 10.1021/acs.analchem.0c01201 32927955

[179] Tseng, P. et al. Functional, RF-trilayer sensors for tooth-mounted, wireless monitoring of the oral cavity and food consumption. *Adv. Mater*. **30**, e1703257 (2018). 10.1002/adma.201703257 29572979

